# Nanoscale Porphyrin-Based Metal–Organic Frameworks for Enhanced Radiotherapy–Radiodynamic Therapy: A Comprehensive Review

**DOI:** 10.3390/pharmaceutics17070883

**Published:** 2025-07-04

**Authors:** Bin Gong, Qiuyun Zhang, Yijie Qu, Xiaohua Zheng, Weiqi Wang

**Affiliations:** 1 The People’s Hospital of Danyang, Affiliated Danyang Hospital of Nantong University, Danyang 212300, China; 2 School of Pharmacy, Nantong University, Nantong 226001, China

**Keywords:** porphyrin, metal–organic frameworks, radiotherapy, radiodynamic therapy, cancer therapy

## Abstract

The phototherapeutic applications of porphyrin-based nanoscale metal–organic frameworks (nMOFs) are limited by the poor penetration of conventional excitation light sources into biological tissues. Radiodynamic therapy (RDT), which directly excites photosensitizers using X-rays, can overcome the issue of tissue penetration. However, RDT faces the problems of low energy conversion efficiency, requiring a relatively high radiation dose, and the potential to cause damage to normal tissues. Researchers have found that by using some metals with high atomic numbers (high Z) as X-ray scintillators and coordinating them with porphyrin photosensitizers to form MOF materials, the excellent antitumor effect of radiotherapy (RT) and RDT can be achieved under low-dose X-ray irradiation, which can not only effectively avoid the penetration limitations of light excitation methods but also eliminate the defect issues associated with directly using X-rays to excite photosensitizers. This review summarizes the relevant research work in recent years, in which researchers have used metal ions with high Z, such as Hf^4+^, Th^4+^, Ta^5+^, and Bi^3+^, in coordination with carboxyl porphyrins to form MOF materials for combined RT and RDT toward various cancer cells. This review compares the therapeutic effects and advantages of using different high-Z metals and introduces the application of the heavy atom effect. Furthermore, it explores the introduction of a chemodynamic therapy (CDT) mechanism through iron coordination at the porphyrin center, along with optimization strategies such as oxygen delivery using hemoglobin to enhance the efficacy of these MOFs as radiosensitizers. This review also summarizes the potential of these materials in preclinical applications and highlights the current challenges they face. It is expected that the summary and prospects outlined in this review can further promote preclinical biomedical research into and the development of porphyrin-based nMOFs.

## 1. Introduction

The structural diversity and facile tunability of MOFs have enabled their widespread investigation across various fields, including gas adsorption, chemical sensing, catalysis, and drug delivery [1,2,3]. Porphyrin-based nMOF materials can effectively prevent the aggregation of hydrophobic porphyrin molecules, making them highly promising as carrier materials for biomedical applications of porphyrin photosensitizers [4,5]. Early research primarily focused on using visible light in the range of 400–680 nm to excite porphyrin photosensitizers, generating reactive oxygen species (ROS) to oxidize biomacromolecules within cancer cells, thereby suppressing cancer cell proliferation [6,7,8]. However, during in vivo treatments, researchers found that the tissue penetration depth of visible light is typically less than 1 cm and that this light is easily absorbed by biological tissues [9]. These limitations necessitate higher doses of photosensitizers, stronger excitation light sources, or the restriction of treatment to superficial tumor cells, significantly limiting the biomedical applications of porphyrin-based nMOF materials.

With continuous advancements in nanoscience technology and medical techniques, researchers have recognized radiotherapy as a highly effective cancer treatment modality [10,11,12]. Radiotherapy using X-rays or γ-rays can directly target DNA within cancer cells, causing strand breaks and structural damage [10,13,14]. Additionally, X-rays can interact with H_2_O or H_2_O_2_ to generate hydroxyl radicals, which are highly oxidative and capable of inhibiting cancer cell proliferation [15,16,17,18]. However, conventional radiotherapy relying solely on X-ray irradiation often faces challenges such as side effects from high doses and low energy conversion efficiency [19]. Therefore, developing low-dose X-ray-activated radiotherapy methods has become an urgent need [20,21,22,23]. Researchers have found that combining X-ray excitation with porphyrin-based nMOFs can fully leverage the deep tissue penetration of X-rays and the advantages of porphyrin-based photosensitizers in RDT [24,25,26,27]. MOF materials can be prepared through the direct coordination of high-Z metals (e.g., Hf, Bi) with porphyrin photosensitizers [28,29]. Under X-ray irradiation, these materials not only directly act on H_2_O or H_2_O_2_ to generate hydroxyl radicals but also utilize the photoelectron transfer effect of high-Z metals (e.g., Hf, Au, Pt) to activate porphyrin photosensitizers, producing singlet oxygen and other ROS, further enhancing cancer cell suppression [28,30,31]. Compared to standalone radiotherapy, this approach achieves desirable therapeutic effects with lower X-ray doses, effectively avoiding systemic side effects of radiotherapy [28]. Thus, the combination of porphyrin-based MOFs with X-rays for RT-RDT holds great potential for biomedical applications [26,27].

Research on RT and RDT has remained a major focus in recent years. In 2020, Ni et al. provided an overview of recent advances in the application of nMOFs as nanosensitizers for PDT, CDT, RT, and radiotherapy–radiodynamic therapy (RT–RDT), highlighting their role in enhancing cancer immunotherapy through effective antigen delivery as nanocarriers for cancer vaccines [32]. A year later, in 2021, Xu et al. reviewed the design principles and post-synthetic functionalization strategies of two-dimensional nanoscale metal–organic layers, with a focus on their use in RT–RDT combination therapy, as well as their potential as platforms for ratiometric sensing, imaging, and targeted drug delivery [33]. In 2022, He et al. presented a comprehensive summary of the latest developments in X-ray-induced photodynamic therapy, discussing its synergistic relationship with RDT and conventional RT [15]. Also in 2022, Pan et al. compiled a review on nanostructure-mediated approaches aimed at achieving precision radiotherapy, emphasizing various nanotechnology-based strategies to improve tumor targeting and reduce collateral damage to healthy tissues, while also addressing current limitations and future directions for clinical translation [34]. RT based on certain high-atomic-number metals can directly destroy cancer cells through physical damage, whereas RDT, which incorporates organic photosensitizer molecules, acts on cells through chemical reactions, such as the generation of ROS. Utilizing a single nanoplatform, such as nMOFs, to achieve combined RT-RDT therapy may represent a highly promising approach for cancer treatment. Therefore, more detailed reviews are needed to summarize recent research efforts focused on nMOF-based nanoplatforms that enable simultaneous RT-RDT combination therapy. These reviews should outline the synthetic strategies employed in fabricating such materials and compare their therapeutic performance, providing an optimized selection framework to identify the most promising candidates for clinical applications.

**Figure 1:**
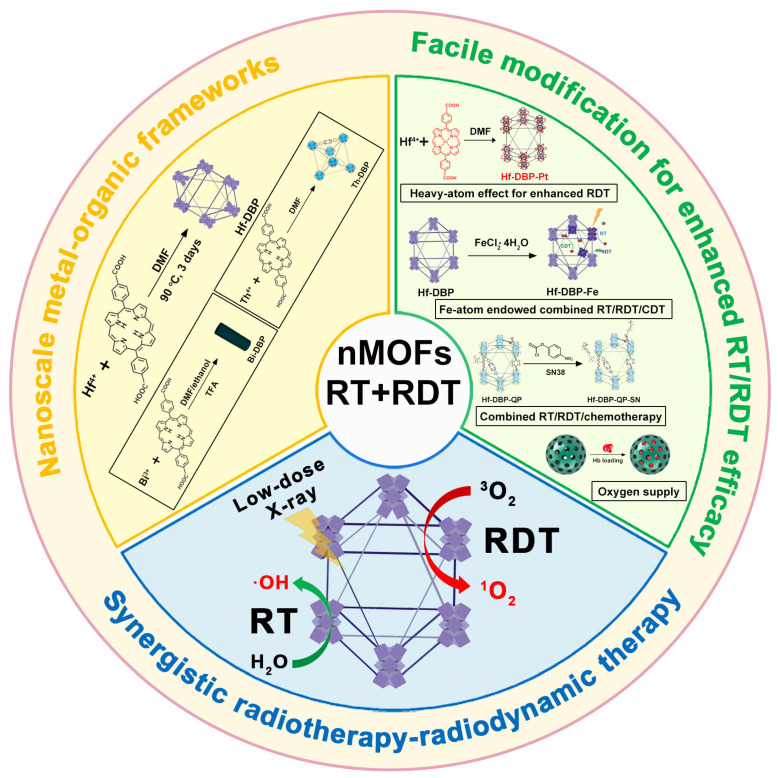
Synthesis routes of Hf-DBP, Bi-DBP, and Th-DBP nMOF materials, along with material modification strategies to enhance therapeutic efficacy for combined RT and RDT treatment.

This review summarizes recent advances in porphyrin-based nMOF materials for RT-RDT combination therapy under X-ray or γ-ray excitation (Figure 1). MOF materials prepared from heavy atom metals and porphyrin ligands exhibit several unique advantages in combined RT and RDT. First, the inherent porous structure of MOFs facilitates the diffusion of ROS, enabling better oxidative effects [35,36]. Second, the high proportion of heavy atom metals and porphyrin ligands in MOF structures allows the efficient delivery of radiosensitizers and porphyrin molecules. Finally, the modifiability and diversity of MOF materials enable the design and synthesis of multifunctional MOFs, potentially leading to unexpected anticancer effects [37,38,39,40]. This review not only expands the biomedical applications of porphyrin-based nMOF materials but also provides new preclinical data references for candidate drugs aimed at effectively suppressing malignant cancer cells.

## 2. Design Rationale and Advantages of Porphyrin–MOFs for Combined RT-RDT

**Figure 2:**
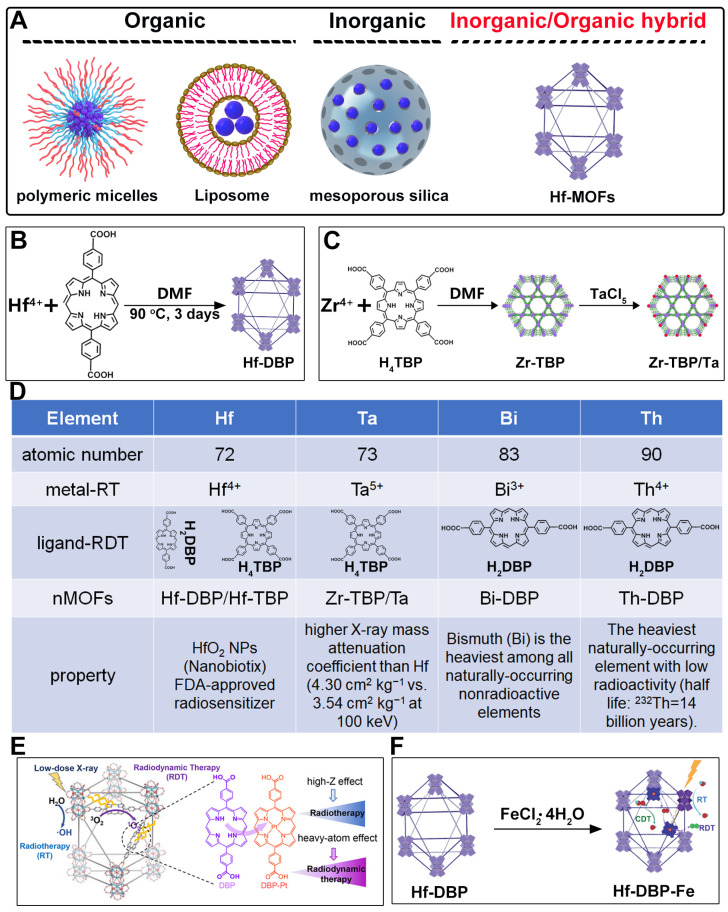
(**A**) Schematic illustration of high-Z-metal-loaded carriers, including organic carriers (polymers, liposomes), inorganic carriers (mesoporous silica nanoparticles), and inorganic/organic hybrid materials (metal–organic frameworks). (**B**) Schematic illustration of the synthesis of Hf-DBP. (**C**) Schematic illustration of the synthesis of Zr-TBP/Ta. (**D**) Atomic numbers of four heavy atoms (Hf, Ta, Bi, and Th), structures of porphyrin ligands required for the formation of four MOFs, and characteristics of the prepared nanomaterials in radiotherapy (RT). (**E**) Influence of porphyrin ligands with and without a central Pt element on RT/RDT. (**F**) Synthetic route of the Hf-DBP-Fe MOF material.

Metal nanoparticles have demonstrated significant potential in biomedical applications due to their unique physicochemical properties. However, their potential in vivo toxicity remains a major concern [41]. To enhance their stability and biocompatibility, various carrier materials are commonly employed (Figure 2A). Traditional carriers mainly include organic materials, such as PEGylated polymers (e.g., DSPE-PEG) or liposomes [42,43,44,45], as well as inorganic materials like mesoporous silica (Figure 2A) [46,47]. These carriers have proven effective in reducing toxicity and improving cellular internalization efficiency. Nevertheless, most of these carrier systems adopt a spherical morphology. Studies have shown that, based on Monte Carlo simulation results, layered structure-shaped nanoparticles outperform cubic and spherical morphologies in terms of radiation dose enhancement [48]. Moreover, conventional polymeric or silica-based carriers are typically inert and lack additional biological functionalities, which limits their capacity for synergistic therapeutic effects. Additionally, the potential long-term toxicity of mesoporous silica poses a barrier to its further clinical translation [49].

Therefore, the development of novel and multifunctional metal carrier materials is of great research significance. MOFs, as hybrid inorganic/organic materials, offer distinct advantages in this field. First, through the adjustment of synthesis conditions such as solvent composition or catalysts (e.g., acid), MOFs can be tailored into nanoscale layered structures, which are beneficial for enhancing radiation dose deposition. Second, their intrinsic porous structure facilitates the generation of hydroxyl radicals during radiotherapy, thereby enhancing therapeutic efficacy. Third, MOFs can be constructed using high-Z metals and porphyrin-based photosensitizers, enabling a synergistic combination of radiotherapy and radiodynamic therapy, offering a more effective strategy for treating refractory malignant tumors. Furthermore, the periodic and ordered architecture of MOFs effectively prevents the aggregation of both metal nanoparticles and photosensitizers, making them more responsive to irradiation. Finally, the structural diversity and facile functionalization capability of MOFs provide broad opportunities for the development of multifunctional therapeutic platforms incorporating various high-Z metals.

The preparation of MOF materials through the coordination of heavy atoms with porphyrin ligands typically involves two approaches: (1) Heavy atoms, such as Hf, can be directly used as metal nodes that participate in the formation of MOF structures (Figure 2B) [28]. This method achieves a higher content of Hf atoms, providing more effective radiosensitization. (2) Alternatively, MOF materials can be prepared first, followed by the introduction of high-Z metals through co-doping (Figure 2C) [50]. This approach not only endows MOF materials with RDT functionality but also expands the variety of radiosensitizers. Studies have shown that the ability of heavy atom metals to effectively attenuate and absorb X-ray energy is strongly correlated with their atomic numbers. This review summarizes research efforts on the preparation of MOF materials using four heavy atom metals—Hf (atomic number: 72) [28], Ta (atomic number: 73) [50], Bi (atomic number: 83) [29], and Th (atomic number: 90) [51]—with dicarboxyl or tetracarboxyl porphyrin ligands for combined RT and RDT to suppress cancer cell proliferation (Figure 2D). Furthermore, this review compares the advantages of these four heavy atom metals and their potential for future preclinical applications.

HfO_2_ nanoparticles (NPs) based on Hf have already received clinical approval, making research on combined RT and RDT treatments using Hf elements worthy of further time and effort (Figure 2D) [52,53,54,55]. Ta has attracted significant attention due to its higher X-ray mass attenuation coefficient compared to Hf, and its excellent performance as a CT contrast agent and photoacoustic imaging agent keeps related research at the forefront (Figure 2D) [56,57,58,59,60]. Bi not only exhibits superior radiosensitization compared to Hf but is also the heaviest non-radioactive element in nature, potentially offering new opportunities for radiotherapy (Figure 2D) [18,41,61,62,63,64,65,66]. Th is a naturally occurring heavy atom metal; despite its low-level radioactivity, its extremely long half-life makes related research highly promising for bioapplication (Figure 2D) [67,68,69,70,71,72].

In addition to enhancing the combined therapeutic effects of RT and RDT by using metals with higher atomic numbers, the structure of porphyrin ligands can also be optimized. Platinum (Pt)-coordinated porphyrin derivatives have been found to exhibit significantly enhanced photodynamic and photocatalytic performance [73,74,75,76,77,78]. Therefore, the rational design and synthesis of Pt-coordinated porphyrin materials with specific functional groups for the preparation of nMOFs are expected to further enhance their ability to generate ROS under photoexcitation or X-ray irradiation. For example, Pt-coordinated porphyrins can be used to form MOFs, where the heavy atom effect of Pt significantly enhances the efficiency of singlet oxygen generation during RDT (Figure 2E) [79]. Alternatively, porphyrin ligands can coordinate with Fe^3+^ ions, whose redox capability enables MOF materials to react with excess H_2_O_2_ in cancer cells to generate O_2_, thereby enhancing RDT efficacy [80]. This process also produces more hydroxyl radicals, enabling CDT effects (Figure 2F). These diverse structural designs not only optimize therapeutic outcomes but also further expand the application scope of MOF materials prepared with heavy atom metals and porphyrins.

This review comprehensively summarizes research efforts on porphyrin-based MOFs for combined RT and RDT treatments. To further highlight and compare the structural components of these different MOFs, the size and morphology of the prepared materials, and their mechanisms for inhibiting cancer cells, detailed information on the MOFs discussed in this review is compiled in Table 1. Through a comparison of the morphology and size of these materials and the types of cancer cells inhibited by them, it is evident that porphyrin-based MOFs not only exhibit excellent performance but also possess significant potential for further application expansion.

## 3. Hf-Based nMOFs for Enhanced RT-RDT and Synergistic Immunotherapy

**Figure 3:**
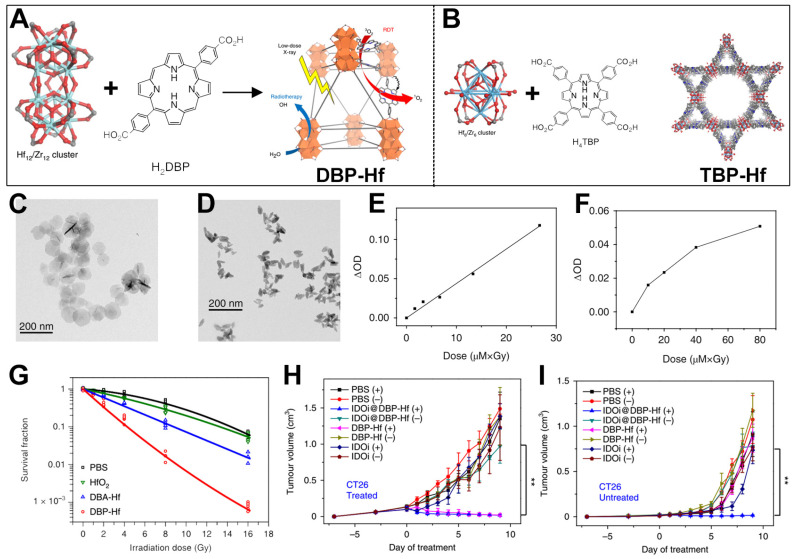
(**A**) Synthesis schematic of DBP-Hf nMOFs. (**B**) Synthesis schematic of TBP-Hf nMOFs. (**C**) TEM diagrams of DBP-Hf. (**D**) TEM diagrams of TBP-Hf. (**E**) ^1^O_2_ generation ability of DBP-Hf. (**F**) ^1^O_2_ generation ability of TBP-Hf. (**G**) Survival fractions of various groups (PBS, HfO_2_, DBA-Hf, or DBP-Hf). (**H**) Tumor volume changes after various treatments toward CT26 cells. ** *p* < 0.01. (**I**) Volume changes from untreated tumors of CT26. Reproduced with permission from [28]. Copyright (2018), Springer Nature.

Zr and Hf are elements in Group IVB of the periodic table [83,84,85]. Due to the influence of lanthanide contraction, the atomic and ionic radii of hafnium are very close to those of zirconium, resulting in extremely similar chemical properties. In chemical reactions, hafnium also mainly exhibits an oxidation state of +4. Both Zr and Hf elements can coordinate with porphyrin ligands to form MOF materials [86,87,88,89,90,91]. Differently from Zr, Hf has a relatively high Z of 72, which enables it to absorb X-ray photons for radiotherapy and RDT treatment mediated by porphyrin photosensitizer ligands [28]. Based on this, Lu et al. prepared two types of nMOF materials and used the pore structures of the nMOFs to load IDO inhibitors, achieving the goal of combined treatment of breast cancer and colorectal cancer through X-ray-excited RT, RDT, and checkpoint blockade therapy [28]. The specific structures of the two nMOFs are as follows: 5,15-bis(p-benzoic acid)porphyrin-Hf (DBP-Hf) (Figure 3A) and 5,10,15,20-tetrakis(p-benzoic acid)porphyrin-Hf (TBP-Hf) (Figure 3B). Subsequently, the authors analyzed the morphology and properties of the prepared nMOFs. Transmission electron microscopy (TEM) results showed that DBP-Hf presented a disk-like shape, while TBP-Hf showed a rod-like shape. Then, the authors determined the ability of DBP-Hf and TBP-Hf to generate ^1^O_2_ under X-ray irradiation using the 4-nitro-N,N-dimethylaniline assay (Figure 3E,F). As shown in Figure 3E, the ability of DBP-Hf to generate ^1^O_2_ showed a certain linear relationship with the product of the nMOF and X-ray doses. Similar results indicated that as the doses of nMOF and X-ray increased, the change in the optical density of 4-nitroso-N,N-dimethylaniline at 439 nm in the TBP-Hf detection system increased, suggesting an increase in the generated ^1^O_2_ (Figure 3F). After that, the authors measured the survival response of TUBO cells under different irradiation doses through a colony-forming assay. To highlight the excellent combined radiotherapy and RDT effects of the prepared DBP-Hf, the authors also used HfO_2_ NPs and DBA-Hf nMOFs prepared from Hf^4+^ and non-photosensitizer ligands as controls (Figure 3G). The experimental results showed that under the same irradiation dose, the radiosensitization effect of DBP-Hf was better than that of HfO_2_. The authors speculated that this was because the porosity of nMOFs was beneficial for the formation and diffusion of free radicals. DBP-Hf had the best radiosensitization effect, which originated from the combined effect of Hf-mediated radiotherapy and porphyrin photosensitizer-mediated RDT. Subsequently, taking DBP-Hf as an example, the authors used its pore structure to load an IDO inhibitor (a widely studied immunomodulatory enzyme) [92] and conducted an inhibition experiment on a bilateral tumor model in mice. As shown in Figure 3H, compared with the group injected with the IDO inhibitor alone, both DBP-Hf and IDOi@DBP-Hf under X-ray irradiation could effectively inhibit the growth of CT26 cancer cells. For distant tumors, only IDOi@DBP-Hf plus X-ray irradiation could inhibit the growth of cancer cells in the experimental group through the systemic immune response (Figure 3I). Immunotherapy is a widely researched strategy proven highly effective for cancer treatment [93,94,95]. The porous structure of nMOFs provides significant advantages for the implementation of combination therapies involving immunotherapy. Moreover, the work provides an important reference for the preclinical application of porphyrin-based nMOFs. Differently from PDT with limited penetration depth, this combined RT-RDT treatment method can effectively treat tumors in deep-seated tissues. Even with a 4.5 cm thick beef block in between, X-rays could still effectively activate the Hf–porphyrin nMOF materials, resulting in a cancer cell death rate of over 50%.

## 4. Ta-Zr-Co-Doped nMOFs for Combined RT and RDT

**Figure 4:**
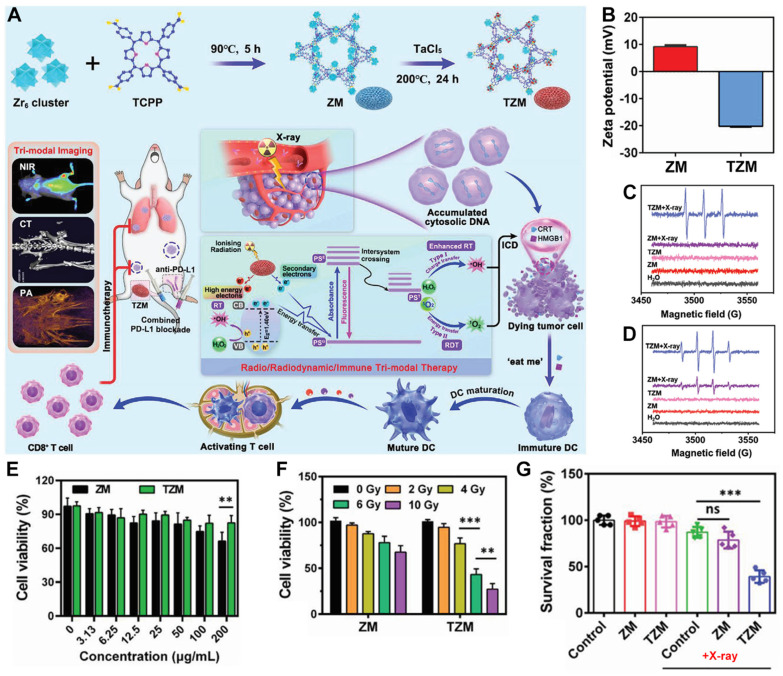
(**A**) Preparation schematic of TZM and its anticancer mechanism toward metastatic osteosarcoma. (**B**) Zeta potential of ZM and TZM. (**C**,**D**) ESR spectra for (**C**) ^1^O_2_ generation ability of various groups by electron spin resonance. (**D**) ·OH generation ability of various groups by electron spin resonance. (**E**) Cell viability of K7M2 cells after addition of ZM and TZM. (**F**) Cell viability of K7M2 cells after addition of ZM and TZM under various X-ray irradiation intensities. (**G**) Survival fraction of K7M2 cells after various treatments. ns: no significant difference; ** *p* < 0.01, *** *p* < 0.001. Reproduced with permission from [50]. Copyright (2023), Wiley-VCH Verlag.

In addition to directly using Hf with a high Z to form nMOFs with porphyrins for combined RT-RDT treatment, the method of doping can also be adopted to introduce atoms with high Z into conventional nMOF structures [50]. This approach not only enriches the types of porphyrin-based nMOFs for combined RT-RDT treatment but also provides a new method for combining metals that cannot form nMOFs directly. For instance, Li et al. first synthesized a Zr-TCPP MOF (referred to as ZM) (Figure 4A) [50]. They then doped ZM with TaCl_5_ to prepare TZM, a Ta-doped Zr-TCPP MOF (Figure 4A). The prepared TZM composite could effectively generate RT and porphyrin-mediated RDT functions under X-ray irradiation, thereby inhibiting the growth and metastasis of osteosarcoma by inducing immunogenic cell death (Figure 4A). Subsequently, the authors found that the surface charge of ZM was 9.2 mV, which changed to −20.2 mV for TZM. This result is consistent with the reported negative charge of Ta NPs and also proves that the Ta element was successfully doped into the Zr-TCPP MOF. Electron spin resonance (ESR) test results showed that only TZM containing high-atomic-number elements could generate ^1^O_2_ (Figure 4C), and TZM produced significantly more ·OH than ZM (Figure 4D), indicating the effective sensitization ability of the Ta element. Since ZM does not contain high-atomic-number elements, it cannot effectively exert the functions of RT and RDT. Then, the authors used the CCK-8 assay (a commonly used method for evaluating the cytotoxicity of drug molecules) [96,97,98,99] to verify the inhibitory effects of ZM and TZM on K7M2 cells. Without X-ray irradiation, both ZM and TZM showed good biocompatibility (Figure 4E). As the X-ray intensity increased from 0 Gy to 10 Gy, it was found that the survival rate of K7M2 cells treated with TZM decreased significantly, which was due to the radiosensitization effect of Ta (Figure 4F). Thereafter, the authors co-incubated K7M2 cells with PBS, ZM, and TZM and measured their survival fractions. The results showed that under X-ray irradiation, only the survival fraction of the TZM experimental group was less than 50% (Figure 4G). This result further proves that the radiosensitization ability of the Ta element can effectively endow the Zr-TCPP MOF with RT-RDT functions. This system provides a reference for the further biomedical application of the Ta metal. It also offers a new example for the clinical application of radiosensitizers. However, it is important to note that this system incorporates two metallic elements, Zr and Ta. While each of these metals may exhibit good biocompatibility when used individually, the potential synergistic toxic effects arising from their coexistence within the same nanoparticle system have not been thoroughly investigated. This lack of understanding could represent a significant barrier to their clinical application.

## 5. Th-Based nMOFs for Combined RT and RDT

**Figure 5:**
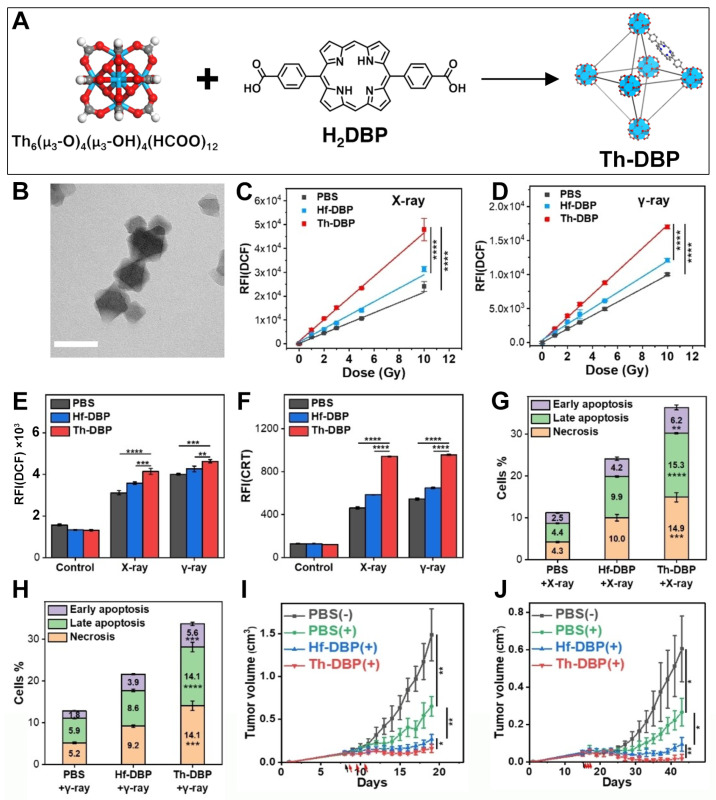
(**A**) Preparation procedure of Th-DBP. (**B**) TEM diagram of Th-DBP. (**C**,**D**) ROS generation ability of PBS, Hf-DBP, and Th-DBP detected by DCFH-DA probe after X-ray (**C**) or γ-ray (**D**) treatment. (**E**,**F**) Comparative results of ROS generation ability (**E**) and surface CRT expressions (**F**) of CT26 cells after treatment with Hf-DBP and Th-DBP. (**G**,**H**) Proportions of CT26 cells undergoing early apoptosis, late apoptosis, and necrosis following exposure to Hf-DBP or Th-DBP, subsequently irradiated with 2 Gy of X-ray (**G**) or 4 Gy of γ-ray (**H**). (**I**,**J**) Alterations in the volumes of CT26 tumors (**I**) and Panc02 tumors (**J**) post treatment with Th-DBP or Hf-DBP and subsequent X-ray irradiation. * *p* < 0.1, ** *p* < 0.01, *** *p* < 0.001, **** *p* < 0.0001. Reproduced with permission from [51]. Copyright (2022), Wiley-VCH Verlag.

The doping method can be used to introduce high-atomic-number radiosensitizers into nMOFs prepared from common metals. However, this method cannot guarantee the maximum loading of radiosensitizers. As radiosensitizers for clinical trials, HfO_2_ NPs have been approved by the US FDA, widely studied, and used as a control material. There are also many types of Hf-based nMOF materials [89,100,101,102,103]. To further develop nMOF materials with stronger radiosensitization effects, some researchers have found that other metals with more energy deposition under X-rays can be used to form MOF radiosensitizers with porphyrin ligands. For example, Th is a naturally occurring metal element with relatively low radioactivity. Its high Z makes its use in the construction of MOF materials possible. Based on this, Xu et al. used the solvothermal method to prepare Th-DBP MOF (Figure 5A) [51]. TEM characterization results showed that a Th-DBP MOF presented a nano-octahedral morphology with a diameter of ~80 nm (Figure 5B). Thereafter, the authors used DCFH-DA as a probe to detect the ability of Hf-DBP and Th-DBP MOF materials to generate ROS under X-ray and γ-ray irradiation. As shown in Figure 5C,D, Th-DBP had a stronger ability to generate ROS than Hf-DBP under both X-ray and γ-ray irradiation. Moreover, as the ray intensity increased, the amount of ROS generated by Th-DBP also significantly increased. The comparison results of the fluorescence intensity of DCF also indicated that Th-DBP was a stronger radiosensitizer than Hf-DBP (Figure 5E). To further study the treatment results of Th-DBP and Hf-DBP in cell and animal experiments, the authors used PEG polymer materials to modify the surfaces of the two MOF materials. After modification, the two MOF materials had similar cell uptake behaviors. Then, the authors evaluated the expression of calreticulin on the surface of CT26 cancer cells induced by Th-DBP and Hf-DBP. As shown in Figure 5F, due to the stronger ROS-generating ability of Th-DBP under ray irradiation, its ability to induce CRT expression was also significantly improved. Next, the authors conducted cytotoxicity tests. As shown in Figure 5G,H, due to the enhanced combined RT/RDT effect, Th-DBP showed a better effect in killing CT26 cancer cells than Hf-DBP under both X-ray and γ-ray irradiation. Then, the authors further verified the superiority of Th-DBP over Hf-DBP through in vivo experiments. As shown in Figure 5I, under X-ray irradiation, Th-DBP could effectively inhibit the proliferation of CT26 tumor cells in the BALB/c mouse model, showing certain advantages over the Hf-DBP radiosensitizer material. Similarly, as shown in Figure 5J, Th-DBP could also effectively inhibit the proliferation of Panc02 cancer cells in the C57BL/6 mouse model, indicating the effectiveness of Th-MOFs as radiosensitizers for the treatment of malignant tumors. This system not only provides new methods for the development of porphyrin-based MOF materials through the development of new high-atomic-number metals to form porphyrin-based MOF materials but also provides new candidate radiosensitizers for the treatment of colon cancer and pancreatic cancer. However, it should be pointed out that although Monte Carlo simulations indicate superior performance of Th over Hf, there are uncertainties in practical applications. Firstly, Th is a relatively rare metallic element with limited availability and challenges in maintaining consistent purity, which may hinder the scalable fabrication of related materials. Secondly, variations in the hypoxic microenvironments among different solid tumors, as well as discrepancies between clinical radiotherapy equipment and the simulation conditions used in the study, may affect the translational relevance. Finally, tumor heterogeneity across different cancer types could lead to significant variations in therapeutic outcomes. Therefore, extensive experimental data are still required to support the clinical application of such materials.

## 6. Bi-Based nMOFs for Combined RT and RDT

**Figure 6:**
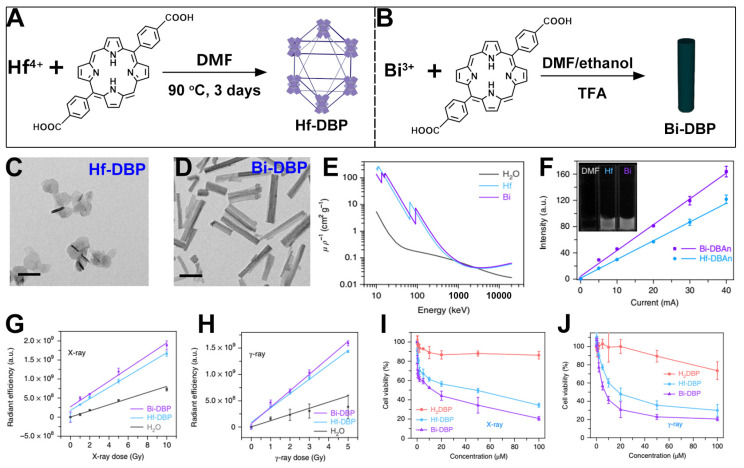
(**A**) Preparation schematic of Hf-DBP. (**B**) Preparation schematic of Bi-DBP. (**C**,**D**) TEM diagrams of (**C**) Hf-DBP and (**D**) Bi-DBP (scale bar = 100 nm). (**E**) Mass attenuation coefficients of three experimental groups (H_2_O, Hf, and Bi). (**F**) Optical images (shown in inset) along with linear fitting of radioluminescence intensities against X-ray tube current for Bi-DBP and Hf-DBP. (**G**,**H**) Enhancement of APF fluorescence in Bi-DBP and Hf-DBP compared to H2O at equivalent concentrations of 20 μM, under X-ray (**G**) and γ-ray (**H**) irradiation. (**I**,**J**) Cell viability of TRAMP-C2 cells after treatment with H_2_DBP, Hf-DBP, and Bi-DBP under X-ray (**I**) and γ-ray (**J**) irradiation. Reproduced with permission from [29]. Copyright (2022), Springer Nature.

The effect of metals as radiosensitizers is related to their atomic numbers [104,105,106,107,108,109]. To ensure biomedical applications, the metals must also be non-radioactive elements. Currently, it is well known that Bi is the naturally occurring element with the highest atomic number among non-radioactive elements [110,111]. Therefore, using Bi as a metal cluster to construct MOF structures has great potential in the development of a radiosensitizer [112,113]. For example, Ni et al. first prepared Hf-DBP as a comparative reference (Figure 6A) [29]. Then, using the solvothermal method, Bi-DBP MOFs were prepared with Bi^3+^ and p-dicarboxyphenyl porphyrin as the building materials (Figure 6B). After that, the authors conducted property tests and functional characterizations of the two MOF materials. As shown in Figure 6C,D, Hf-DBP presented a disk shape as reported in the literature, while Bi-DBP showed a nanorod shape with a diameter * length of approximately 20 nm * 180 nm. Through testing, it was found that the K-edge energy of the Bi element at 90 keV is higher than that of the Hf element at 65 keV. This result indicates that the Bi element is more suitable for enhancing the secondary radiation of photons generated by a linear accelerator used in clinical settings (Figure 6E). In addition, the authors found through testing that the radiation signal of Bi-FBAn is 1.38 times that of Hf-DBAn, which shows that the Bi element has a higher X-ray absorption and energy transfer efficiency (Figure 6F). Next, the authors verified the ability of the two MOF materials to generate ·OH. As shown in Figure 6F,G, under both X-ray and γ-ray irradiation, the fluorescence detected by aminophenyl fluorescein (APF) for the Bi-DBP MOF was stronger than that for the Hf-DBP MOF. This indicates that Bi-based MOFs can exhibit a stronger sensitization effect. Then, the authors examined the cytotoxic effects of Hf-DBP and Bi-DBP on TRMP-C2 cells. As shown in Figure 6I,J, under an X-ray voltage with a dose of 2 Gy or ^60^Co γ-ray irradiation, the cell survival rate of the group treated with Bi-DBP was lower than that of the group treated with Hf-DBP. This result shows that under the same conditions, Bi-DBP MOFs can exert a stronger radiotherapy effect. Nevertheless, these studies are still at the laboratory level. In clinical practice, factors such as cost and the production yield of metal elements may need to be considered. Furthermore, although free heavy metals such as Au and Pb are generally associated with significant toxicity, the Bi metal itself is typically considered to have good biocompatibility [114,115,116,117]. However, it is important to note that Bi-MOFs may exhibit instability in the presence of phosphate or under other tumor microenvironmental conditions, leading to their gradual degradation and the release of soluble Bi^3+^ ions. These soluble Bi^3+^ ions may subsequently bind to biomacromolecules in vivo, affecting their distribution and metabolism. Therefore, efficient excretion of Bi^3+^ ions remains a key challenge for the clinical application of such materials.

## 7. Hf-Based nMOFs: Boosting RT and RDT via Heavy Atom Effect

**Figure 7:**
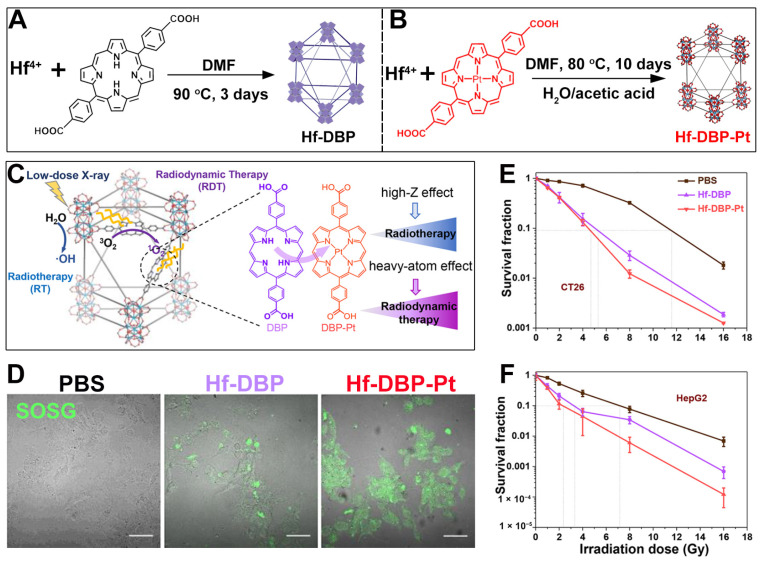
(**A**) Preparation procedure of Hf-DBP. (**B**) Preparation procedure of Hf-DBP-Pt. (**C**) Schematic of Pt metalation boosting energy deposition and intersystem crossing, enhancing RT-RDT. (**D**) ^1^O_2_ generation ability after addition of Hf-DBP and Hf-DBP-Pt using SOSG assay. (**E**,**F**) Survival fraction after the addition Hf-DBP and Hf-DBP-Pt into CT26 (**E**) and HepG2 (**F**) cells under X-ray irradiation. Reproduced with permission from [79]. Copyright (2021), American Chemical Society.

In addition to using metal elements with larger atomic numbers such as Th and Bi as participants in the construction of porphyrin-based nMOFs, which can enhance the radiotherapy effect of the sensitizer materials, there are also some methods to enhance the efficacy of RDT. It is well known that the heavy atom effect can enhance the ability of organic photosensitizers to generate ^1^O_2_ by promoting intersystem crossing between the singlet state and the triplet excited state of the photosensitizer [78,118,119,120,121]. Building on this, Guo et al. first synthesized a Hf-DBP MOF as a control (Figure 7A) [79]. They then prepared a platinum(II)-metallated DBP-Pt coordination complex and incorporated it into the Hf-DBP framework to obtain a Hf-DBP-Pt MOF (Figure 7B). The heavy atom effect of Pt^2+^ enhances the porphyrin photosensitizer’s ability to generate ^1^O_2_, as shown in Figure 7C. Subsequently, the authors used SOSG as a probe to detect the ability of Hf-DBP and Hf-DBP-Pt to generate ^1^O_2_ under the same X-ray irradiation conditions. As shown in Figure 7D, the green fluorescence of the group treated with Hf-DBP-Pt was significantly stronger than that of the Hf-DBP group. This result directly proves the enhancing effect of the heavy atom effect on porphyrin-mediated RDT. Then, the authors directly evaluated the radiosensitization effects of Hf-DBP and Hf-DBP-Pt on cancer cells in vitro. As shown in the results of the colony formation experiments in Figure 7E,F, for both CT26 cells and HepG2 cells, under the same X-ray dose, the survival fractions of cancer cells treated with Hf-DBP-Pt were lower. This directly proves the enhanced combined effect of RT-RDT induced by the heavy atom effect. This system endows the porphyrin-based nMOF with an enhanced ability to generate ^1^O_2_ by introducing the heavy atom Pt, thus achieving an enhanced combined effect of RT/RDT.

## 8. Hf-Based nMOFs: Boosting RT and RDT via Self-Oxygen-Carrying Function

**Figure 8:**
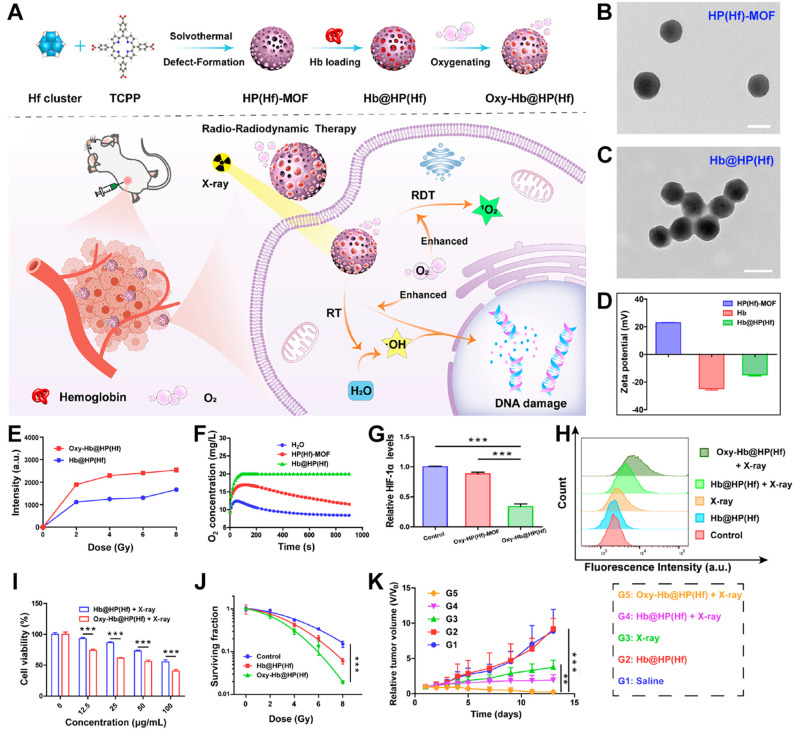
(**A**) Preparation schematic of Hb@HP(Hf) nanosensitizer and its anticancer mechanism. (**B**) TEM picture of HP(Hf)-MOF. (**C**) TEM picture of Hb@HP(Hf). (**D**) ζ-potentials of Hb, HP(Hf)-MOF, and Hb@HP(Hf) in solution. (**E**) ^1^O_2_ generation ability of two groups under different doses of X-ray. (**F**) O_2_ concentration changes in three groups after oxygenation. (**G**) The changes in relative HIF-1α levels for three groups. (**H**) Intracellular ROS generation ability after various treatments detected by flow cytometry analysis. (**I**) Cell viabilities of CT26 cells after incubation with Hb@HP(Hf) and Oxy-Hb@HP(Hf) under X-ray irradiation. (**J**) Survival fraction of CT26 cells after various treatments. (**K**) Tumor volume changes for five groups (G1–G5). ** *p* < 0.01, *** *p* < 0.001. Reproduced with permission from [81]. Copyright (2023), American Chemical Society.

The efficacy of PDT, sonodynamic therapy, and the combined RT-RDT treatment method may be hindered by the hypoxic environment of solid tumors [122,123]. Therefore, it is necessary to improve the hypoxic microenvironment of solid tumors while carrying out these treatment methods for enhanced therapeutic efficacy [124]. There are many ways to alleviate the hypoxic state of solid tumors [125,126]. Various strategies can be employed to increase intracellular O_2_ concentration, including the introduction of mitochondrial respiration inhibitors or the use of Pt NPs as catalysts to react with excess intracellular H_2_O_2_ [127,128,129,130,131,132,133]. Combination therapy strategies can also effectively overcome the limitations imposed by hypoxic environments on ROS-based treatment approaches [134,135,136]. In addition, proteins that transport oxygen, such as hemoglobin (Hb) in human red blood cells, can be used to coat and modify the materials. In the combined RT-RDT treatment protocol, using Hb to modify nMOF radiosensitizers can effectively improve the treatment effect. Based on this, Zhao et al. first prepared a Hf-MOF (Hb(Hf)-MOF) (Figure 8A) [81], which, due to its positive charge and porous structure, can adsorb hemoglobin (Hb) to form Hb@HP(Hf). Upon exposure to oxygen, the material enables Hb to transport oxygen, which is then released inside cells to alleviate hypoxia. Under X-ray excitation, Hb@HP can combine with water molecules, enhancing the formation of ·OH and exerting an enhanced RT effect. The increase in oxygen concentration helps the X-ray activation of porphyrin photosensitizers to generate ^1^O_2_, thus exerting an enhanced RDT effect and causing damage to the DNA of cancer cells (Figure 8A). As shown in Figure 8B, the prepared Hf-TCPP MOF presents a spherical shape and is well-dispersed. Figure 8C shows that the morphology of Hb@HP NPs obtained after modification with Hb remains essentially unchanged. As shown in Figure 8D, the surface of the Hf-TCPP MOF is charged, and the Hb@HP MOF obtained after being modified by negatively charged Hb molecules shows a negative charge similar to that of Hb. This result proves that the method of simply stirring and loading Hb molecules onto the surface of the Hf-TCPP MOF is effective. Afterward, the authors used SOSG as a probe to determine the ability of Hb@HP and Oxy-Hb@HP to generate ^1^O_2_ under the same X-ray irradiation. As shown in Figure 8E, after loading oxygen, the ability of the material to generate ^1^O_2_ under X-ray irradiation is significantly enhanced. Then, the authors used an oxygen detector to detect the change in the oxygen concentration of three groups of solutions, namely, H_2_O, Hf-MOF, and Hb@HP, after bubbling oxygen for 15 min. As shown in Figure 8F, the oxygen concentration of the Hb@HP experimental group was maintained, which proves that Hb can indeed normally perform the function of transporting oxygen. The results of hypoxia-inducible factor detection also indicate that the Hf-TCPP MOF modified by Hb (Hb@HP) can significantly improve the hypoxic state of cells (Figure 8G). The authors used flow cytometry to detect the fluorescence intensity of DCF in cells (DCF-DA can be oxidized by ^1^O_2_ to generate fluorescent DCF; the stronger the fluorescence, the more ^1^O_2_ is generated). The experimental results in Figure 8H show that after modifying Hf-MOF with O_2_-transporting Hb, an enhanced RDT effect can indeed be exhibited under X-ray irradiation. Following that, the authors evaluated the cytotoxicity of the prepared materials. Under the same concentration and X-ray irradiation, the survival rate of CT26 cells in the Oxy-Hb@HP group was significantly lower than that in the group without oxygen transportation (Figure 8I). The results of the colony survival experiment are consistent with those of the cytotoxicity test (Figure 8J). These results all indicate that improving the O_2_ concentration helps to enhance the combined effect of RT-RDT. Based on these results, the authors conducted an in vivo experiment to inhibit CT26 tumors. As shown in Figure 8K, the G5 group (Oxy-Hb@HP) exerted the most beneficial effect in inhibiting the volume of cancer cells under X-ray irradiation. This system achieves an enhanced combined effect of RT-RDT by modifying the MOF radiosensitizer with oxygen-transporting hemoglobin, providing an important reference for research on new radiosensitizers.

## 9. Hf-DBP-Fe nMOFs for RT-RDT Combined with CDT

**Figure 9:**
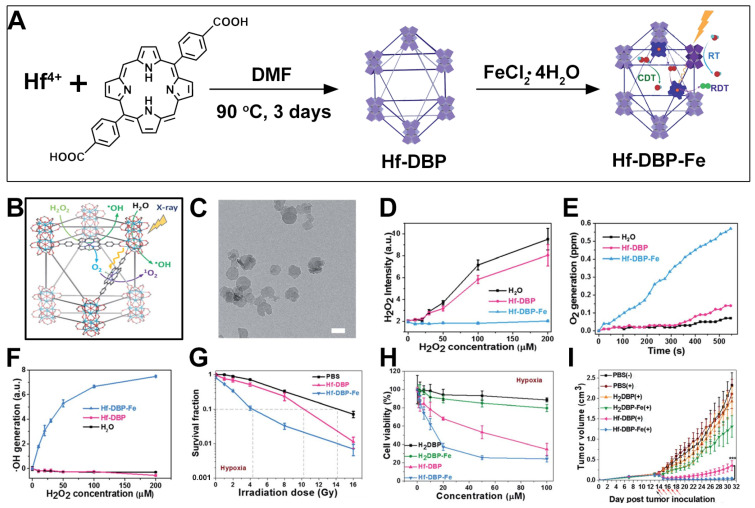
(**A**) Preparation procedures of Hf-DBP-Fe nMOFs. (**B**) CDT and RT-RDT mechanism schematic of Hf-DBP-Fe nMOFs. (**C**) TEM picture of Hf-DBP-Fe. (**D**) H_2_O_2_ concentration changes for various groups detected by a H_2_O_2_ fluorescence kit. (**E**) O_2_ generation ability of three groups detected by an O_2_ sensor. (**F**) ·OH generation ability of three groups. (**G**) Clonogenic assay assessing radioenhancement of Hf-DBP-Fe, Hf-DBP, and PBS under hypoxic conditions with X-ray irradiation. (**H**) Cell viability of MC38 cells after incubation with H_2_DBP, H_2_DBP-Fe, Hf-DBP, or Hf-DBP-Fe (X-ray irradiation + hypoxic conditions). (**I**) Tumor volume changes in MC38 tumor-bearing mice after various treatments. Reproduced with permission from [80]. Copyright (2020), The Royal Society of Chemistry.

In addition to the utilization of hemoglobin, using variable valence metals such as Fe^3+^/Fe^2+^ or Mn^4+^/Mn^2+^ as catalysts to react with the excessive H_2_O_2_ in cancer cells to generate O_2_ is also a very effective solution for addressing hypoxic tumors [137]. On the other hand, the porphyrin molecules in porphyrin-based MOF materials can interact with variable valence metals through the coordination of nitrogen elements on the pyrrole ring. Based on this, Lin et al. synthesized a Hf-DBP MOF and further functionalized it with the Fe element to obtain Hf-DBP-Fe (Figure 9A) [80]. The authors found that the Fe element could exhibit catalase-like activity. It could not only catalyze the generation of O_2_ from H_2_O_2_ but also exert a CDT effect under X-ray irradiation, enabling combined RT/RDT/CDT for a stronger antitumor effect (Figure 9B). The TEM test results showed that Hf-DBP-Fe presented a nanosheet morphology similar to that of Hf-DBP (Figure 9C). The H_2_O_2_ kit showed that Hf-DBP-Fe could effectively catalyze and reduce the concentration of H_2_O_2_ (Figure 9D), thereby generating O_2_ to improve the hypoxic state (Figure 9E). The APF probe also demonstrated that Hf-DBP-Fe could cause H_2_O_2_ to generate ·OH (Figure 9F). The colony formation experiments also proved that Hf-DBP-Fe could exhibit a stronger inhibitory effect on MC38 cells with a lower cell survival fraction under hypoxic conditions (Figure 9G). The cytotoxicity test revealed that both Hf-DBP and Hf-DBP-Fe showed good biocompatibility without X-ray irradiation. However, under X-ray irradiation, even under hypoxic conditions, both Hf-DBP and Hf-DBP-Fe could exhibit cytotoxicity (Figure 9H). But due to the catalase-like activity and CDT effect exerted by Hf-DBP-Fe formed with the participation of the Fe element, it showed a stronger inhibitory effect on MC38 cells with a lower IC50 value (Figure 9H). Subsequently, the authors verified the inhibitory effect on MC38 cancer cells in tumor-bearing mice. As shown in Figure 9I, under the same drug administration concentration condition, the group treated with Hf-DBP-Fe + X-ray irradiation achieved the best antitumor effect. Through the utilization of the structural characteristics of the porphyrin molecules in the MOF in coordination with the Fe element, the porphyrin-based MOF is endowed with more therapeutic functions and realizes the combined function of hypoxia relief and enhanced RT/RDT/CDT.

## 10. Mixed-Ligand nMOFs for RT-RDT Combined with Chemotherapy

**Figure 10:**
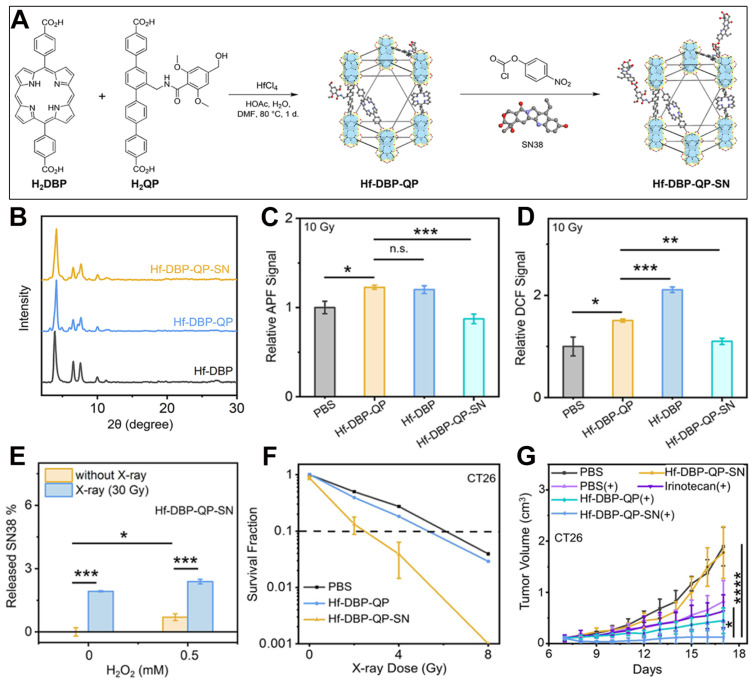
(**A**) Preparation procedures of Hf-DBP-QP and Hf-DBP-QP-SN. (**B**) PXRD results of Hf-DBP, Hf-DBP-QP, and Hf-DBP-QP-SN. (**C**) ·OH generation ability and (**D**) ROS generation ability under X-ray irradiation. (**E**) Release efficiency of SN38 from Hf-DBP-QP-SN under X-ray irradiation, with and without H_2_O_2_. (**F**) Survival fraction of CT26 cells after treatment with Hf-DBP-QP and Hf-DBP-QP-SN under X-ray irradiation. (**G**) Volume changes in CT26 tumors after various treatments. (ns: no significant difference, * *p* < 0.1, ** *p* < 0.01, *** *p* < 0.001, **** *p* < 0.0001). Reproduced with permission from [82]. Copyright (2024), American Chemical Society.

In addition to strategies such as using metals with higher atomic numbers as MOF metal clusters, applying the heavy atom effect, and alleviating the hypoxic microenvironment of cancer cells to achieve enhanced RT-RDT efficacy, combining RT-RDT with treatment methods like chemotherapy can also enhance the therapeutic effect [138,139,140]. Differently from RT-RDT, most chemotherapy drug molecules are likely to cause systemic toxicity [141,142,143,144]. Therefore, it is of great significance to develop chemotherapy activated by special stimuli such as pH, hypoxia, and ROS, enabling it to act at the lesion site [145,146,147,148,149]. Through combination with the hydroxyl radicals generated during the RT-RDT process, the creation of a hydroxyl radical-activated release system can be attempted to develop a combined treatment protocol of RT-RDT and chemotherapy. Based on this, Zhen et al. incorporated quaterphenyl dicarboxylic acid derivative (H2QP) into the Hf-DBP structure to form Hf-DBP-QP [82]. Then, the 7-ethyl-10-hydroxycamptothecin (SN38) molecule was loaded to obtain Hf-DBP-QP-SN (Figure 10A). The bond between H2QP and the SN38 molecule can respond to the hydroxyl radicals generated during the RT-RDT process, thus releasing SN38 for chemotherapy. The PXRD test results proved that the formation of the MOF with the participation of H_2_QP and the subsequent loading of the SN38 molecule had little impact on the crystalline structure of Hf-DBP (Figure 10B). Thereafter, the authors evaluated the generation of hydroxyl radicals from the material by detecting the change in the APF signal intensity. As shown in Figure 10C, the total amount of hydroxyl radicals generated by Hf-DBP-QP was similar to that of Hf-DBP, but the amount of hydroxyl radicals generated by the Hf-DBP-QP-SN group decreased to a certain extent. This was because the generated hydroxyl radicals reacted with the chemical bonds formed by QP-SN, consuming some hydroxyl radicals and proving that SN38 could be effectively released (Figure 10C). Then, the authors evaluated the total amount of ROS generation by detecting the fluorescence change in DCF generated from the DCFH-DA probe. As shown in Figure 10D, under an X-ray irradiation of 10 Gy, the total amount of ROS generated by Hf-DBP-QP was less than that of the Hf-DBP group. This was because the doping of QA molecules reduced the amount of porphyrin molecules. Hf-DBP-QP-SN generated the least amount of total ROS due to its response to hydroxyl radicals. Since X-rays can promote the generation of hydroxyl radicals from H_2_O_2_, the authors conducted an experiment on the release of SN38 under the condition of adding additional H_2_O_2_ (Figure 10E). X-rays can stimulate water and H_2_O_2_ to generate hydroxyl radicals. Therefore, the release of SN38 molecules was most effective when both X-rays and hydrogen peroxide were present (Figure 10E). Following that, the authors evaluated the radiosensitization effect of the materials prepared in this system through a colony experiment. As shown in Figure 10F, under the excitation of the same X-ray dose, the survival fraction of CT26 cells in the group treated with Hf-DBP-QP-SN was significantly lower than that in the Hf-DBP-QP group. This result indicates that the introduction of the SN38 chemotherapy molecule significantly enhanced the combined RT/RDT treatment effect of Hf-DBP. Then, the authors determined the inhibitory effect of Hf-DBP-QP-SN on CT26 cancer cells in mice under X-ray irradiation. As shown in Figure 10G, compared with multiple other control groups, the tumor volume of the mice in the group treated with Hf-DBP-QP-SN + X-rays was the smallest. This result proves the effectiveness of introducing the SN38 chemotherapy molecule into this system for achieving an enhanced inhibitory effect on malignant cancer cells. This system also provides a unique design method for the combination of RT/RDT mediated by porphyrin-based nMOF materials and responsive chemotherapy.

## 11. Comparative Study of nMOFs Derived from Hf, Ta, Bi, and Th

**Figure 11:**
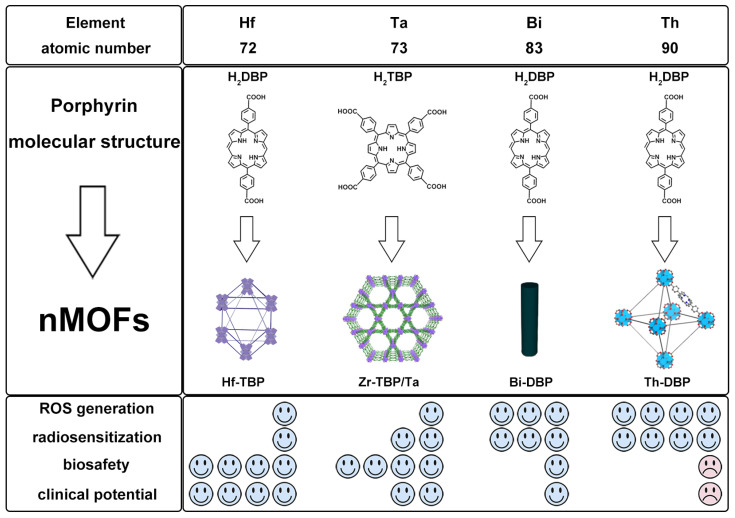
Comparative study of nMOFs derived from Hf, Ta, Bi, and Th.

Although nMOFs based on the four metals have demonstrated excellent combined antitumor efficacy in RT-RDT at both cellular and murine levels, a more in-depth comparison of their ROS generation capacity and safety profiles is still needed to further advance their clinical translation. To address this, we present a systematic evaluation of these nMOFs in Figure 11, focusing on four key aspects: ROS generation efficiency, radiosensitization performance, toxicity, and potential for clinical translation.

In terms of ROS generation efficiency, although Ta has a higher atomic number and greater X-ray mass attenuation coefficient than Hf (and HfO_2_), it can only be incorporated into MOFs via doping. As a result, the amount of Ta in the MOF structure is limited by the initial feed ratio, making it difficult to confirm whether Ta-based MOFs exhibit stronger ROS generation capability than Hf-MOFs [50]. Experimental data indicate that the Bi-MOF shows significantly enhanced ROS generation compared to the Hf-MOF, while the Th-MOF demonstrates the highest ROS production efficiency [51]. Regarding radiosensitization performance, the sensitization effect of the four metals increases progressively with an increasing atomic number, from Hf, Ta, and Bi to Th. According to the literature data, under identical conditions, the radiosensitization ability of the Bi-MOF is 1.12 times that of the Hf-MOF (2.08 vs. 1.86) [29], while that of the Th-MOF reaches 1.45 times (1.77 vs. 1.22) [51]. In terms of biosafety and clinical application potential, the Hf-MOF exhibits the most promising translational prospects due to the excellent biocompatibility of Hf and the clinical use of HfO_2_ as a radiosensitizer. Tantalum (Ta) is also widely used in biomedical applications such as orthopedics and is typically non-radioactive in nature. However, studies on Ta-based materials remain limited, and more experimental data are needed to support their clinical translation. Bismuth (Bi) is considered a material with negligible radiotoxic risk and high radiosensitization efficiency. However, Bi-based MOFs often require functional modifications to mitigate potential toxicity. Thorium (Th), as a weakly radioactive element, requires careful safety evaluation for its clinical application. Due to concerns related to radioactivity, the clinical translation of Th-based MOFs may face significant challenges.

## 12. Conclusions

Malignant tumors have long been one of the most serious diseases threatening human survival, leading to the development and in-depth investigation of various treatment modalities [150,151,152,153,154,155]. Numerous studies have shown that combination therapy is a more effective strategy compared to single-mode treatments [156,157,158]. The structural tunability and diverse types of nMOFs make the implementation of combination therapy significantly more feasible. This review summarizes the relevant research progress in recent years on the combined effect of RT/RDT on cancer cells, which is generated by porphyrin-based nMOF materials under the excitation of X-rays or γ-rays. As porous materials (nMOFs) with excellent biocompatibility, nMOFs’ structural features facilitate the diffusion of ^1^O_2_ generated by X-ray activation and the realization of therapeutic effects. The structural diversity and easy modifiability of porphyrin-based MOFs endow them with potential for the development and application of nanomedicines excited by low-dose X-rays.

However, RT-RDT using materials similar to Hf–porphyrin MOFs still faces numerous challenges before clinical application. Firstly, the porphyrin molecules involved in coordination can only be synthesized through complex organic synthesis procedures in the laboratory, and the yield is relatively low. Secondly, some of the research work summarized in this review attempts to use metals with high Z such as Ta, Th, and Bi to prepare nMOF materials, but this may remain at the laboratory research stage. These metals with high Z may face the problem of high costs, which makes it difficult for them to be widely applied in clinical settings. Next, large amounts of detailed research data on various pharmacological and toxicological indicators, such as the in vivo metabolism and tissue distribution of nMOFs prepared from these metals with high Z, are still needed to support and prove the feasibility of their clinical application. Ultimately, the clinical translation of these materials will require collaborative efforts from researchers across multiple disciplines, including materials scientists, chemists, and medical experts, to ensure successful practical implementation. Solving these existing problems will contribute to the further promotion of the biomedical applications of porphyrin-based MOF materials.

## Figures and Tables

**Table 1:** Synthesis of porphyrin-based nMOFs and their integrated RT/RDT mechanisms for enhanced antitumor applications.

Material	Structure		Properties and Therapeutic Advantages	Ref.
	Porphyrin	nMOF		
Hf-DBP, Hf-TBP	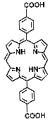	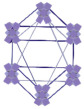	Hf-DBP, nanoplate size = 72.3 nm, 630 nm, RT (^1^O_2_)-RDT (·OH); Hf-TBP, fusiform, size = 72.7 nm, 650 nm, RT (^1^O_2_)-RDT (·OH), RT/RDT for CT26	[28]
Zr-TBP/Ta, Zr/Ta co-doped MOFs (TZM)	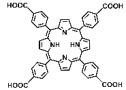	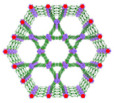	120 nm in width and 300 nm in length, RT (^1^O_2_)-RDT (·OH), RT/RDT for metastatic osteosarcoma	[50]
Th-DBP, Th-DBP@PEG	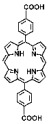	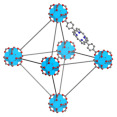	nano-octahedron, 80 nm, RT (^1^O_2_)-RDT (·OH), RT/RDT for CT26 and Panc02 cells	[51]
Bi-DBP	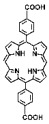	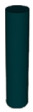	nanorod, diameter (~20 nm) and length (~180 nm), RT (^1^O_2_)-RDT (·OH), RT/RDT for TRAMP-C2 cells	[29]
Pt—heavy atom effect, Hf-DBP-Pt	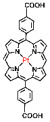	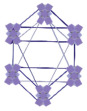	nanoplate, diameter (~100 nm) and thickness (~8 nm), RT (^1^O_2_)-RDT (·OH), RT/RDT for CT26 and HepG2 cells	[79]
Hf-TBP, HP(Hf)-MOF, Hb@HP(Hf)	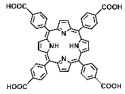	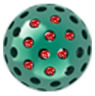	spherical structure, 200 nm, O_2_-carrying, RT (^1^O_2_)-RDT (·OH), RT/RDT for 4T1 and CT26 cells	[81]
Fe(III)-mediated CDT, Hf-DBP, Hf-DBP-Fe	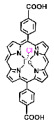	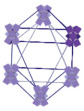	nanoplate, ~100 nm, CDT (O_2_ generation, ·OH), RT (^1^O_2_)-RDT (·OH), RT/RDT for MC38 cells	[80]
Hf-DBP-QP, Hf-DBP-QP-SN	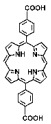	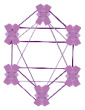	nanoplate, diameter (~120 nm) and thickness (~20 nm), chemotherapy, RT (^1^O_2_)-RDT (·OH), RT/RDT for CT26 cells	[82]

DBP (5,15-di(p-benzoato)porphyrin); TBP (5,10,15,20-tetra(p-benzoato)porphyrin); Hb (hemoglobin); QP (quaterphenyl dicarboxylic acid); SN (7-ethyl-10-hydroxycamptothecin).
